# Positive association between serum apolipoprotein M levels and hepatitis B virus DNA load in HBeAg-negative chronic hepatitis B

**DOI:** 10.1186/s12944-016-0384-3

**Published:** 2016-12-07

**Authors:** Ting Shen, Wei Min Wu, Wen Han Du, Lin Wang, La Gu He, Li Tan, ZeYou Wang, Ruohong Chen, Min Hu, Ya Ping Ren

**Affiliations:** Department of Laboratory Medicine, The Second Xiangya Hospital, Central South University, Changsha, 410011 Hunan Province China

**Keywords:** Apolipoprotein M, Chronic hepatitis B, HBV DNA load

## Abstract

**Background:**

Hepatitis virus B (HBV) has infected millions of people worldwide. Notably, such infections can be associated with hepatic complications. Levels of apolipoprotein M (apoM), a component of high-density lipoprotein (HDL), are known to be significantly elevated in patients with chronic hepatitis B (CHB). The aim of this study was to investigate the relationship between HBV DNA load in serum and serum apoM levels in patients with CHB.

**Methods:**

A total of 73 HBeAg-negative CHB patients, 50 HBeAg-positive CHB patients, and 79 non-CHB controls were included in the study cohort. The age and body mass index (BMI) of the study participants were matched. Serum levels of apoM and the HBV antigens HBsAg and HBeAg were measured by enzyme-linked immunosorbent assay (ELISA) analysis. Serum levels of alanine aminotransferase (ALT), aspartate transaminase (AST), cholesterol, and triglycerides (TG) were assessed using an automatic biochemical analyzer. Serum HBV DNA levels were quantified by real-time PCR analysis. Data were analyzed by Spearman’s rank correlation coefficient, Pearson correlation coefficient, and multivariate linear regression model (continuous variables), or Student’s *t*-test (mean differences).

**Results:**

Both the HBeAg-negative CHB and HBeAg-positive CHB patient groups exhibited elevated serum levels of apoM. Moreover, serum apoM levels were positively correlated with serum HBV DNA levels in HBeAg-negative CHB patients (*r* = 0.394, *p* < 0.001). Conversely, there was no significant relationship between apoM and HBV DNA levels in the HBeAg-positive CHB group (*r* = 0.197, *p* = 0.170). The median log copies/mL value for HBV DNA (4.00) was considered the cutoff point for the HBeAg-negative CHB group. Notably, a significant number of patients with HBV DNA levels above the cutoff point also had higher serum apoM levels (63.38 ± 29.84 vs. 41.41 ± 21.84; *p* = 0.001).

**Conclusions:**

Our findings reveal that the correlation between serum apoM levels and viral loads may depend on HBeAg status, as serum apoM levels were positively correlated with HBV DNA levels in HBeAg-negative CHB patients. These results suggest that HBeAg may play a role in apoM-related lipid metabolism and anti-inflammatory functions in hepatitis B patients. Thus, our findings may facilitate the clinical management of HBV infection.

## Background

Hepatitis B virus (HBV) infection causes chronic liver disease in >350 million people worldwide and this disease is positively correlated with incidences of liver cirrhosis and hepatocellular carcinoma. Chronic liver diseases can interfere with hepatic metabolism of lipoproteins and apolipoproteins. Moreover, the ongoing replication of HBV in chronic hepatitis B (CHB) induces oxidative stress and is associated with inflammation of the liver [1–3]. Factors that govern viral replication and determine infection outcome remain unclear, but it is known that host factors play a major role in these processes [4].

Apolipoprotein M (apoM) is a 26-kD apolipoprotein that is primarily associated with high-density lipoproteins (HDL); however, a small proportion of this protein also interacts with triglyceride-rich lipoprotein (TGRLP) and low-density lipoprotein (LDL) in the serum. apoM is a member of the lipocalin protein superfamily and is exclusively expressed in hepatocytes and kidney tubular cells [5, 6]. This protein plays a variety of biological functions, e.g., anti-oxidative function [7], anti-inflammatory function [8], promoting pre-β HDL formation [9], and increasing cholesterol efflux from foam cells [10].

Previous studies reported that chronic hepatitis patients exhibit higher serum apoM levels than healthy control subjects [11, 12]. Similarly, HepG2 cells transfected with an infectious HBV clone were found to express significantly higher levels of apoM mRNA and protein than uninfected HepG2 cells in vitro. Moreover, apoM suppressed HBV replication in HepG2 cells [13]. However, the association between apoM expression and HBV replication rate in CHB patients is poorly understood. Therefore, the aim of the present study was to investigate the association between apoM and HBV replication rate to facilitate the management of anti-viral treatment in clinical practice.

## Methods

### Subjects

CHB patients were recruited at the Second Xiangya Hospital (Changsha, Hunan, PR China) between June 2015 and June 2016. From the 200 individuals screened, 73 HBsAg(+) HBeAg(−) CHB patients and 50 HBsAg(+) HBeAg(+) CHB patients were enrolled in this study. The mean age of the patients in the HBsAg(+) HBeAg(−) group was 44 ± 13 years, and the group was comprised of 62 males and 11 females. Meanwhile, the mean age of the patients in the HBsAg(+) HBeAg(+) group was 40 ± 12 years, and the group was comprised of 43 males and 7 females (Table 1). CHB was diagnosed based on serum HBsAg positivity for at least 6 months. The exclusion criteria for CHB were as follows: (1) positive for hepatitis C virus, hepatitis D virus, and human immunodeficiency virus antibodies; (2) cirrhosis and hepatocellular carcinoma; (3) autoimmune hepatitis and alcoholic liver disease; (4) diabetes; (5) immunological disease; (6) cancer; (7) renal disease; (8) heart disease; and (9) antiviral therapy treatment. Lastly, the control group was comprised of 79 age- and sex-matched HBsAg(−) volunteers who underwent physical examination in our hospital. All subjects provided signed Informed Consent Forms and the study protocol was approved by the Second Xiangya Hospital Investigational Review Board.

**Table 1:** Clinical and biochemical characteristics of the patients included in this study

	Control	HBeAg-negative	HBeAg-positive
Subjects, n	79	73	50
Demographics			
Age, years	44 ± 12	44 ± 13	40 ± 12
Sex, % male	87%	85%	86%
Body mass index, kg/m2	22.2 ± 2.2	22.3 ± 2.1	22.1 ± 2.1
Lipids profile			
Triglycerides, mmol/L	1.1 ± 0.41	1.72 ± 0.50*	2.74 ± 0.62^#^&
Total cholesterol, mmol/L	4.4 ± 0.57	3.35 ± 1.33*	4.12 ± 1.21&
HDL-C, mmol/L	1.35 ± 0.21	0.67 ± 0.47*	0.60 ± 0.36^#^
LDL-C, mmol/L	2.86 ± 0.53	1.96 ± 0.89*	2.59 ± 1.10&
Apolipoprotein A, g/L	1.38 ± 0.19	0.67 ± 0.39*	0.62 ± 0.34^#^
Apolipoprotein B, g/L	0.97 ± 0.16	0.99 ± 0.44	1.24 ± 0.34^#^&
Apolipoprotein M, mg/L	13.95 ± 7.11	52.42 ± 28.09*	58.32 ± 27.06^#^
Lipoprotein(a), g/L	102 ± 2.81	20.67 ± 3.21*	26.72 ± 3.73^#^
liver biochemical profile			
Alanine aminotransferase, IU/L	18.25 ± 1.56	99.73 ± 4.50*	216.77 ± 4.17^#^&
Aspartate transaminase, IU/L	20.11 ± 1.25	99.74 ± 2.95*	171.25 ± 2.80^#^&
Log HBV DNA		4.32 ± 2.03	5.61 ± 1.97&

Data are presented as means ± standard deviations. Values for triglyceride, lipoprotein a, ALT, and AST levels were converted into a logarithmic form prior to analysis. *p < 0.05, HBeAg-negative chronic hepatitis B (CHB) compared to the control. ^#^ *p* < 0.05, HBeAg-positive CHB compared to the control. &*p* < 0.05, HBeAg-positive CHB compared to HBeAg-negative CHB

*HDL-C* high-density lipoprotein cholesterol, *LDL-C* low-density lipoprotein cholesterol, *IU* international units

### Blood sampling

Following overnight fasting (12 h) and at least 20 min of rest, blood samples were collected from each subject. After centrifugation at 1200 × g for 5 min, sera were collected, aliquoted, and stored at −80 °C until use.

### Enzyme-linked immunosorbent assay (ELISA) for apoM determinations

Serum apoM levels were measured by sandwich ELISA (Yuan Tai Bio Inc., Changsha, Hunan, People’s Republic of China) analysis. Absorbance was measured at 450 nm, with a background reading at 620 nm, using an ELX-800 absorbance reader (BioTek Instruments, Inc., Winooski, VT, USA). The concentration of apoM in each sample were determined using a standard curve.

### Virological assessment

Serum levels of HBsAg and HBeAg were measured via ELISA analysis using HBV S antigen and HBV E antigen diagnostic kits (Ke Hua Bio Inc., Shanghai, PR China), respectively. HBV DNA was extracted from 200 μL aliquots of serum using a Hepatitis B Viral DNA Quantitative Fluorescence Diagnostic Kit (Sheng Xiang Bio Inc., Changsha, Hunan, PR China), and quantified by real-time polymerase chain reaction (PCR) using an ABI 7000 real-time detection system (Applied Biosystems, Foster City, CA, USA).

### Lipoprotein and liver biochemical assays

Levels of serum triglyceride (TG), total cholesterol (THOL), HDL cholesterol (HDL-C), LDL cholesterol (LDL-C), apolipoprotein A (apoA), apolipoprotein B (apoB), lipoprotein a (Lpa), alanine aminotransferase (ALT), aspartate transaminase (AST), and total bile acid (TBA) were determined using an ARCHITECTc8000 System (Abbott Laboratories, Irving, TX, USA).

### Statistical analyses

Continuous data are expressed as means ± standard deviations. Relationships between continuous variables were tested by Spearman’s rank correlation coefficient, Pearson correlation coefficient, and multivariate linear regression model analyses. Mean differences were compared by Student’s *t*-test. Statistical analyses were performed using SPSS 20.0 (SPSS Statistics, Inc., Chicago, IL, USA) or GraphPad Prism 5.0 (GraphPad Software, La Jolla, CA, USA) software. Two-tailed *p*-values < 0.05 were considered statistically significant.

## Results

### Demographic and clinical characteristics of the study subjects

**Fig. 1:**
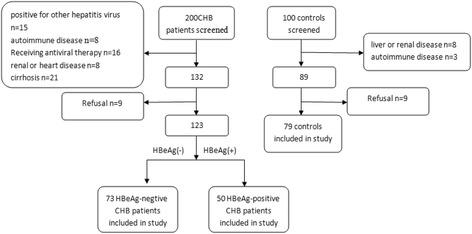
flowchart of the study

Of the 200 CHB patients screened for participation in this study, 73 HBeAg-negative and 50 HBeAg-positive CHB patients were identified and assessed according to the criteria delineated in the Methods section. Additionally, of the 100 control subjects screened for participation, 11 failed the eligibility criteria and 10 refused to participate. As such, 79 control subjects were evaluated in this study (Fig. 1). The demographic and biochemical characteristics of the subjects are summarized in Table 1. Notably, there were no differences between the control, HBeAg-negative, and HBeAg-positive CHB groups in regard to age (44 ± 12, 44 ± 13, and 40 ± 12), gender (87, 85, and 86% male), and BMI (22.2 ± 2.2, 22.3 ± 2.1, and 22.1 ± 2.1), respectively. Conversely, the patients in the HBeAg-negative and HBeAg-positive groups exhibited significantly higher (*p* < 0.05) mean serum apoM levels (52.42 ± 28.09 and 58.32 ± 27.06 mg/L, respectively) than those in the control group (13.95 ± 7.11 mg/L). Meanwhile, there was no significant difference between the HBeAg-negative and HBeAg-positive groups in average apoM serum levels or HBV DNA levels (4.28 ± 2.07 and 5.61 ± 1.97 log copies/mL, respectively).

### Serum liver biochemical profiles of HBeAg-negative CHB patients, HBeAg-positive CHB patients, and normal subjects

**Fig. 2:**
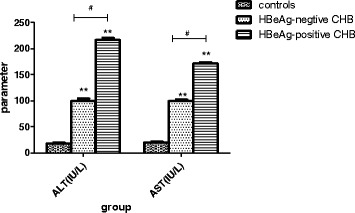
Serum liver biochemical profiles of chronic hepatitis B (CHB) patients. Serum concentrations of liver biochemical markers in CHB patients, grouped according to HBeAg status, and healthy control subjects. *p < 0.05, **p < 0.01, compared to the control group. #*p* < 0.05, between two groups indicated by the horizontal line

As shown in Fig. 2, the average serum ALT and AST levels of the HBeAg-negative CHB patients were 99.73 ± 4.50 IU/L and 99.74 ± 2.95 IU/L, respectively, which were significantly higher than those of the normal subjects (18.25 ± 1.56 IU/L and 20.11 ± 1.25 IU/L, respectively; *p* < 0.01). Notably, however, the ALT and AST levels of the patients in the HBeAg-positive group (216.77 ± 4.17 IU/L and 171.25 ± 2.8 IU/L; *p* < 0.01) were even higher than those observed in the HBeAg-negative group.

### Routine serum lipid levels in HBeAg-negative CHB patients, HBeAg-positive CHB patients, and normal subjects

**Fig. 3:**
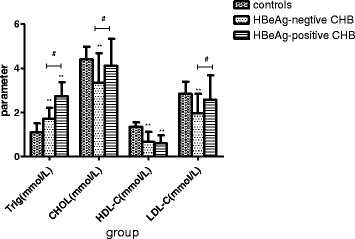
Chronic hepatitis B (CHB) patients exhibit elevated serum triglyceride levels and decreased serum levels of total cholesterol (THOL), high-density lipoprotein cholesterol (HDL-C), and low-density lipoprotein cholesterol (LDL-C). Serum concentrations of THOL, HDL-C, and LDL-C in CHB patients, grouped according to HBeAg status, and healthy control subjects. *p < 0.05, **p < 0.01, compared to the control subjects. #*p* < 0.05, between the two groups indicated by the horizontal line

HBeAg-positive CHB patients exhibited significantly higher (*p* < 0.01 for each) serum Trig levels (2.74 ± 0.62 mmol/L) than those in the HBeAg-negative (1.72 ± 0.50 mmol/L) and control (1.1 ± 0.41 mmol/L) groups (Fig. 3). Moreover, the serum Trig levels of the HBeAg-negative group were also significantly higher than those of the control (*p* < 0.01). Meanwhile, serum HDL-C levels were higher in both the HBeAg-negative and HBeAg-positive CHB patients than in control subjects; however, there was no difference in serum HDL-C levels between the HBeAg-negative and HBeAg-positive CHB groups. Likewise, there were no differences in THOL and LDL-C levels between the HBeAg-positive CHB patients and control subjects. Conversely, the HBeAg-negative CHB patients exhibited elevated levels of THOL and LDL-C, compared to those in the other two groups (Fig. 3).

### Serum apolipoprotein levels in HBeAg-negative CHB patients, HBeAg-positive CHB patients, and normal subjects

**Fig. 4:**
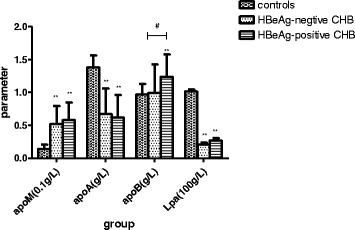
Chronic hepatitis B (CHB) patients exhibit elevated serum apoM and apoB levels and decreased serum apoA and lipoprotein a (Lpa) levels. Serum concentrations of apolipoproteins in CHB patients, grouped according to HBeAg status, and healthy control subjects. *p < 0.05, **p < 0.01, compared to the control group. #*p* < 0.05, between two groups indicated by the horizontal line

While serum apoM levels were higher in both the HBeAg-negative and HBeAg-positive CHB patients than control subjects, these patients exhibited lower serum levels of apoA and Lpa than the controls (Fig. 4). In addition, there were no differences in apoA and Lpa levels between the two CHB groups. Conversely, HBeAg-positive patients exhibited higher serum apoB levels than both the HBeAg-negative patients and control subjects.

### Serum apoM and HBV DNA load in HBeAg-negative and HBeAg-positive CHB patients

As summarized in Table 2, there was no significant difference in the serum levels of apoM between the HBeAg-negative and HBeAg-positive groups (52.42 ± 28.09 vs. 58.32 ± 27.06 mg/L; *p* = 0.248); however, the HBeAg-positive CHB patients exhibited significantly higher HBV DNA loads than those in the HBeAg-negative group (4.28 ± 2.07 vs. 5.61 ± 1.97 log copies/mL; *p* < 0.01).

**Table 2:** Serum apoM levels and hepatitis B virus (HBV) DNA loads in HBeAg-negative and HBeAg-positive chronic hepatitis B (CHB) patients

	HBeAg-negative	HBeAg-positive	*p*-value
Apolipoprotein M, mg/L	52.42 ± 28.09	58.32 ± 27.06	0.248
LogHBV DNA	4.32 ± 2.03	5.61 ± 1.97	0.001

Data are means ± standard deviations. The *p*-values refer to comparisons between the HBeAg-negative and HBeAg-positive groups

### Correlation analysis of serum apoM and HBV DNA levels in HBeAg-negative and HBeAg-positive CHB patients

**Fig. 5:**
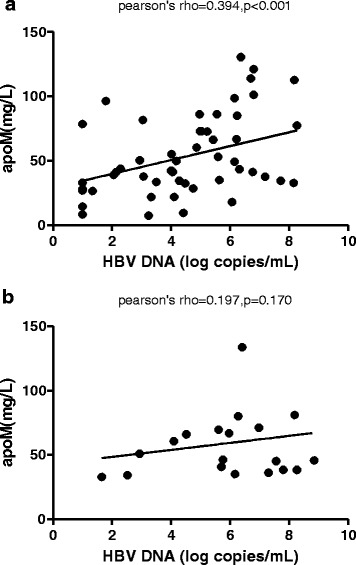
Correlation between serum levels of apoM levels hepatitis B (HBV) DNA. There was a positive correlation between the log10 HBV DNA value and serum apoM levels in (**a**) HBeAg-negative CHB patients (*r* = 0.394; *p* < 0.01), but not in (**b**) HBeAg-positive CHB patients (*r* = 0.197; *p* = 0.170)

Notably, we detected a positive association between serum apoM and serum HBV DNA levels in the HBeAg-negative CHB group (*r* = 0.394; *p* < 0.01), but not in the HBeAg-positive CHB patients group (*r* = 0.197; *p* = 0.170) (Fig. 5).

### Association between serum apoM and HBV DNA levels in HBeAg-negative CHB patients

Using the median value of 4.28 log copies/mL HBV DNA as a cutoff point, we observed that patients with HBV DNA levels exceeding that value also showed elevated levels of apoM (63.38 ± 29.84 vs. 41.41 ± 21.84 mg/L; *p* = 0.001), ALT (213.8 ± 3.4 vs. 45.3 ± 3.9 IU/L; *p* < 0.001) and AST (179.8 ± 2.6 vs. 54.6 ± 2.4 IU/L; *p* < 0.001), respectively (Table 3). Moreover, multivariate regression analysis, using log HBV DNA as a continuous dependent variable and apoM, HDL-C, LDL-C, CHOL, TG, apoA, apoB, age, sex, BMI, AST, ALT, and TBA as independent variables, detected a statistically significant positive association between log HBV DNA levels and serum apoM levels (*p* < 0.001; β = 0.029) (Table 4).

**Table 3:** Association of clinical findings of chronic hepatitis B with HBV DNA levels

	HBV DNA < median*	HBV DNA > median*	*p*-value
Subjects, n	36	37	
Demographics			
Age, years	47 ± 12	42 ± 12	0.109
Sex, % male	84	86	0.364
Body mass index, kg/m^2^	22.2 ± 2.0	22.4 ± 2.4	0.487
Lipid profile			
Triglycerides, mmol/L	1.68 ± 0.51	1.77 ± 0.52	0.817
Total cholesterol, mmol/L	3.51 ± 1.31	3.19 ± 1.34	0.308
HDL-C, mmol/L	0.76 ± 0.48	0.58 ± 0.44	0.099
LDL-C, mmol/L	2.07 ± 0.97	1.86 ± 0.82	0.326
Apolipoprotein A, g/L	0.74 ± 0.37	0.61 ± 0.41	0.177
Apolipoprotein B, g/L	0.93 ± 0.45	1.05 ± 0.43	0.241
Apolipoprotein M, mg/L	41.41 ± 21.84	63.38 ± 29.84	0.001
Lipoprotein(a), g/L	30.27 ± 2.64	14.32 ± 3.41	0.005
Liver biochemistry profile			
Alanine aminotransferase, IU/L	45.3 ± 3.9	213.8 ± 3.4	<0.001
Aspartate transaminase, IU/L	54.6 ± 2.4	179.8 ± 2.6	<0.001

HBV DNA levels were categorized as higher or lower than the median log copies/mL (4.23 log copies/mL). Values for triglycerides, lipoprotein (a), alanine aminotransferase, and aspartate transaminase were converted into logarithmic form before analysis. *p* < 0.05 was considered statistically significant

*HDL-C* high-density lipoprotein cholesterol, *LDL-C* low-density lipoprotein cholesterol, *IU* international units

**Table 4:** Multivariate regression analysis

	Beta	Standardized Beta	*p*-value
Factor correlation			
with Log HBV DNA			
apoM	0.029	0.397	<0.001
apoA	0.011	0.002	0.996
apoB	0.085	0.018	0.969
HDL-C	−0.268	−0.151	0.881
LDL-C	0.966	0.416	0.181
TG	0.485	0.296	0.382
THOL	−0.824	−0.528	0.053
Age	−0.001	−0.004	0.976
Sex	−1.033	−0.192	0.056
BMI	0.001	0.003	0.967
ALT	0.01	0.149	0.285
AST	0.02	0.22	0.169
TBA	−0.001	−0.041	0.775

*apoM* apolipoprotein M, *apoA* apolipoprotein A, *apoB* apolipoprotein B, *HDL-C* high-density lipoprotein cholesterol, *LDL-C* low-density lipoprotein cholesterol, *TG* triglycerides, *THOL* total cholesterol, *BMI* body mass index, *ALT* alanine aminotransferase, *AST* aspartate transaminase, *TBA* total bile acid. *p* < 0.05 was considered statistically significant

## Discussion

In this study, we measured the serum levels of apoM and lipids in HBeAg-negative CHB, HBeAg-positive CHB, and control subjects, and screened for associations between serum apoM levels and HBV DNA load. Indeed, the key finding of our study was a significant positive correlation in vivo between serum apoM and HBV DNA levels in HBeAg-negative patients. These results are consistent with and extend previous findings that CHB patients exhibit elevated serum apoM levels [11, 12].

In this study, CHB patients exhibited lower serum levels of HDL-C and apoA than the control subjects. In contrast, these patients also presented with higher serum levels of apoM, which primarily interacts with HDL-C, than the controls. Elevated apoM levels were also reported in cases of chronic inflammatory disease and decreased in acute inflammatory disease [14, 15]. The potential mechanisms underlying the anti-inflammatory activities and immune-modulatory properties of apoM have been previously characterized [16–18], and apoM is known to be widely distributed within the pool of serum lipoproteins under inflammatory conditions [19, 20]. In chronic inflammatory disease, apoM functions as an innate protective factor that is up-regulated to resist inflammatory effects over long periods. Recently studies showed that apoM could limits endothelial and vascular inflammation though sphingosine 1-phosphate-sphingosine 1-phosphate receptor 1 pathway (S1P-SIP1) [21, 22]. Furthermore, Wang et al. [18] showed that apoM contributed to the maintenance of CD4^+^ T lymphocytes or benefited for modifying T lymphocyte subgroups in murine spleen. Indeed, apoM-sphingosine 1-phosphate (apoM-S1P) was shown to contribute to innate and adaptive immunity by directly interacting with bone marrow lymphocyte progenitors and inhibiting their proliferation via activation of the S1P1/ERK pathway [17]. We proposed that apoM-S1P may play a role in resisting chronic inflammation in CHB patients. What’s more, it may be used as a potential helpful agent in CHB patients.

The pronounced replication of HBV during CHB has been linked to the development of cirrhosis and hepatocellular carcinoma [23, 24]. Notably, this replication also seems to be affected by host factors. Previous studies showed that HBV enhances apoM mRNA and protein expression, and that elevated levels of apoM suppress HBV protein expression and viral replication in HepG2 cells. However, these studies found no correlation between HBV viral load and apoM levels in patients with CHB [13]. Conversely, in this study, we found that higher HBV DNA levels were associated with higher serum apoM concentrations in patients with HBeAg-negative, but not HBeAg-positive, CHB. Moreover, while HBV DNA loads were also higher in HBeAg-positive CHB than in the control subjects, there was no significant difference in serum apoM levels between the HBeAg-positive and HBeAg-negative CHB patients. It is therefore conceivable that high HBeAg titers have an inhibitory effect on the HBV DNA-mediated enhancement of apoM expression. Liver is a major organ for the synthesis of lipoproteins and metabolism of lipids. HBV infection disturbs the synthesis of almost all lipoproteins and enzymes envolving in lipid metabolism [25, 26]. It was reported that HBV down-regulated the expression of apoA through affecting ApoA1 promoter activities [27], probably via promotor hypermethylation [28]. Consistent with these findings, our results found that apoA was lower in CHB patients. Previous study showed that serum leptin was elevated in patients with hepatitis B patients [29], and apoM expression was lower in leptin deficient ob/ob mice. After leptin administration, apoM expression was elevated in the liver and kidney of ob/ob mice [30]. We proposed that HBV may increase apoM level through enhancing leptin expression. Several studies have also reported that HBx promote the expression of LXR [31, 32], and that the LXR agonist TO901317 enhances the expression of apoM in Caco-2 cells [33, 34]. Therefore, HBV may mediate the upregulation of apoM via the RXR/LXR pathway. However, the molecular mechanism underlying these phenomena was not analyzed in the present study. As such, future studies are needed to elucidate the molecular effects of HBeAg on apoM expression.

There are several limitations to our present study. First, the number of patients with CHB evaluated herein was relatively small. Further studies with a larger patient cohort are therefore needed to confirm our findings. Another limitation was the imbalanced sex ratio of the cohort; evaluation of an approximately equal number of male and female subjects would provide more conclusive results. Finally, because we did not characterize the HBV genotypes of the infected patients, it is not clear whether the correlation between HBV DNA and apoM levels is dependent on HBV sub-genotypes. Such data would help determine whether apoM could be utilized as a serum marker to facilitate the management of CHB in clinical practice or a potential helpful agent in CHB treatments.

## Conclusions

In summary, we detected a correlation between serum apoM levels and viral loads that may depend on HBeAg status. Specifically, we detected a positive correlation between serum apoM and serum HBV DNA levels in HBeAg-negative, but not HBeAg-positive, CHB patients. To enhance the reliability of our results, we adjusted for age, sex, BMI, ALT, AST, TBA, HDL-C, LDL-C, CHOL, TG, apoA, apoB, and LPa when screening for a correlation between apoM and HBV DNA levels. Our results support an association between apoM levels and HBV replication in HBeAg-negative CHB. Since apoM functions as an anti-inflammatory and immune-modulatory factor, these results suggest that HBeAg may play a role in apoM-related lipid metabolism and anti-inflammatory functions in hepatitis B patients. Thus, our findings may facilitate the clinical management of HBV infection.

## Abbreviations

ALT: Alanine aminotransferase
apoA: Apolipoprotein A
apoB: Apolipoprotein B
apoM: Apolipoprotein M
AST: Aspartate transaminase
BMI: Body mass index
CHB: Chronic hepatitis B
HBV: Hepatitis B virus
HDL-C: High-density lipoprotein cholesterol
LDL-C: Low-density lipoprotein cholesterol
TBA: Total bile acid
TG: Triglycerides
THOL: Total cholesterol
